# Investigating the current knowledge and needs concerning a follow-up for long-term cardiovascular risks in Dutch women with a preeclampsia history: a qualitative study

**DOI:** 10.1186/s12884-020-03179-1

**Published:** 2020-08-24

**Authors:** Tessa E. Dijkhuis, Femke Bloem, Lise A.J. Kusters, Sofie M. Roos, Sanne J. Gordijn, Floor Holvast, Jelmer R. Prins

**Affiliations:** 1 Department of Obstetrics and Gynaecology, University Medical Center Groningen, University of Groningen, Groningen, Netherlands; 2 Department of General Practice, University Medical Center Groningen, University of Groningen, Groningen, Netherlands

**Keywords:** Follow-up, Preeclampsia, Cardiovascular risk, Aftercare, Qualitative study

## Abstract

**Background:**

There is increasing evidence that a history of preeclampsia is an important risk factor for future cardiovascular events. Awareness of this risk could provide opportunities for identification of women at risk, with opportunities for prevention and / or early intervention. A standardized follow-up has not yet been implemented in the north of the Netherlands. The objective of this qualitative study was to explore the opinions and wishes among women and physicians about the follow-up for women with a history of preeclampsia.

**Methods:**

Semi-structured interviews with 15 women and 14 physicians (5 obstetricians, 4 general practitioners, 3 vascular medicine specialists and 2 cardiologists) were performed and addressed topics about knowledge on CVR, current - and future follow-up. Women were approached through the HELLP foundation and their physicians. Physicians were approached by email. The interviews were recorded, typed and coded using ATLAS.ti software. A theoretical-driven thematic analysis was performed.

**Results:**

Women had some knowledge about the association between preeclampsia and the increased CVR, but missed information from their health care providers. Specialists were aware of the association, but the information and advice they provided to their patients was minimal and inconsistent according to themselves. Whereas some general practitioners regarded their own knowledge as limited. There was a clear desire among women for a more extensive follow-up with specific attention to both emotional and physical consequences of preeclampsia. Physicians indicated that they preferred to see a follow up program concerning the CVR at the general practitioner as part of the already existent cardiovascular risk management (CVRM) program.

**Conclusion:**

Women and medical specialists consider it important to improve aftercare for women after a pregnancy complicated by preeclampsia. Introducing these women into the CVRM program at the general practitioner is regarded as a preferred first step. Further research is warranted to establish an evidence-based guideline for the follow-up of these women.

## Background

Preeclampsia is a pregnancy-specific syndrome characterized by the onset of hypertension and proteinuria at more than 20 weeks of gestation [1]. In the Netherlands, approximately 1–3% of pregnancies are complicated by preeclampsia [2]. Increasing evidence shows that a history of preeclampsia represents an important risk factor for future cardiovascular and cerebrovascular events [3–5]. Studies have shown a two-fold increased risk of cardiovascular disease (CVD), and a threefold increased risk of hypertension and heart failure [5–9].

The association between a history of preeclampsia, future hypertension and CVD provides opportunities for the identification of women at risk, early intervention, and prevention [10]. Several large observational studies and meta-analyses have shown that follow-up and early interventions, including lifestyle modifications and increasing awareness, are beneficial to reduce the incidence of CVD in these women [9–12]. In 2011 the American Heart Association added a history of preeclampsia to the existing risk factors for cardiovascular disease and included lifestyle recommendations for these women [13]. However, in clinical practice, this recommendation is not always implemented [8, 12]. Concerning Dutch national guidelines, a Dutch multidisciplinary guideline for cardiovascular risk management (CVRM) after pregnancy-related disorders was constituted in 2014. Based on this meta-analysis of the existing literature a recommendation for follow-up was formulated [8]. This recommendation includes modification of lifestyle and treatment of hypertension, till the age of 50. Above the age of 50, the advice is to follow the national CVRM guidelines. Several other recommendations and guidelines have been published on CVRM after hypertensive disorders of pregnancy (HDP) [14, 15]. These are, however, not uniform in their advice on the timing and content of the follow-up [6, 8, 16].

Although there is considerable evidence that CVRM follow-up for women with a history of preeclampsia is valuable [5, 8, 10]; it has barely been implemented in the north of the Netherlands. This could be due to limited research on the woman and/or physician perspectives on this topic. Little thought has been put into implementation strategies and feasibility in current care providing settings, the awareness amongst women and physicians, as well as their wishes [17, 18].

Therefore, this study aims to define the perspectives of women with a medical history of preeclampsia and their treating physicians on future follow-up program for women with preeclampsia. Moreover, this study aimed to investigate the current knowledge among these women and physicians on the association between preeclampsia and the increased risk of future cardiovascular morbidity and mortality. We conducted semi-structured interviews with women, obstetricians, internal medicine specialists, cardiologists, and general practitioners (GPs). Through assessing their knowledge, identifying barriers for implementation, and evaluating their motivation and wishes, we aimed to formulate recommendations concerning the follow-up for women who have had preeclampsia.

## Methods

### Design

For this study we used a qualitative study design using semi-structured interviews. The interview used in your study was developed for this study (see supplementary file for English translation). Recruitment of participants, conducting and transcribing the interviews and the analysis were performed between January and April 2019. This qualitative study was approved by the Medical Ethical Committee of the University Medical Center Groningen (METc 2019/077). All women and physicians gave written informed consent.

### Participants and recruitment

Eligible women were recruited with the help of the Dutch HELLP foundation. The HELLP foundation is an online platform which gives women the possibility to contact other women who have had the same experience during their pregnancies. The platform provides information about (pre-)eclampsia and the HELLP syndrome, through their website, and organizing information meetings for women. Women were included if they had a medical history of preeclampsia, were between twenty and fifty years of age, and were living in the northern part of the Netherlands. As we did not have access to the medical files, it remains unclear whether all women suffered from preeclampsia or gestational hypertension or HELLP, most women could not recall this themselves. During the study period 27 women responded to our message that was posted on behalf of the HELLP foundation. From these women, two were excluded for not living in the Northern part of the Netherlands and five women withdrew from the study due to personal circumstances. Another 5 women eventually were not invited for an interview due to achieving already enough participants with different patient characteristics [19]. In total fifteen women were invited for an interview. The recruitment of physicians took place at the University Medical Center Groningen, in the department of Gynaecology & Obstetrics, Internal medicine, Cardiology, and in GP offices in the region. Suitable physicians within the region were identified and ten gynaecologists, ten internal medicine specialists and nine GPs were contacted by email. Five gynaecologists specialized in obstetric medicine (obstetricians), four GPs, three internal medicine specialists, and two cardiologists were interviewed. The other doctors were not included as they gave no response or enough doctors were already interviewed to achieve a representative sample.

### Interview

The interviews took place at the participants preferred location. These locations included the UMCG, the participants ´ home, and the GPs practice. The interviews were conducted by two researchers and lasted approximately 20–40 min. The contents of the interviews were created according to existing guidelines for designing semi-structured interviews [20].

The interview was divided into three main categories and the contents were adjusted to the appropriate group of participants. The first category consisted of questions about ‘the current knowledge of women and physicians concerning CVD ‘following preeclampsia’. In the second category, ‘current interventions and follow-up’, the existing practice surrounding the follow-up care was questioned. The third category, ‘future follow-up’, incorporated questions about possibilities and personal preferences regarding the follow-up.

### Data-analysis

**Fig. 1:**
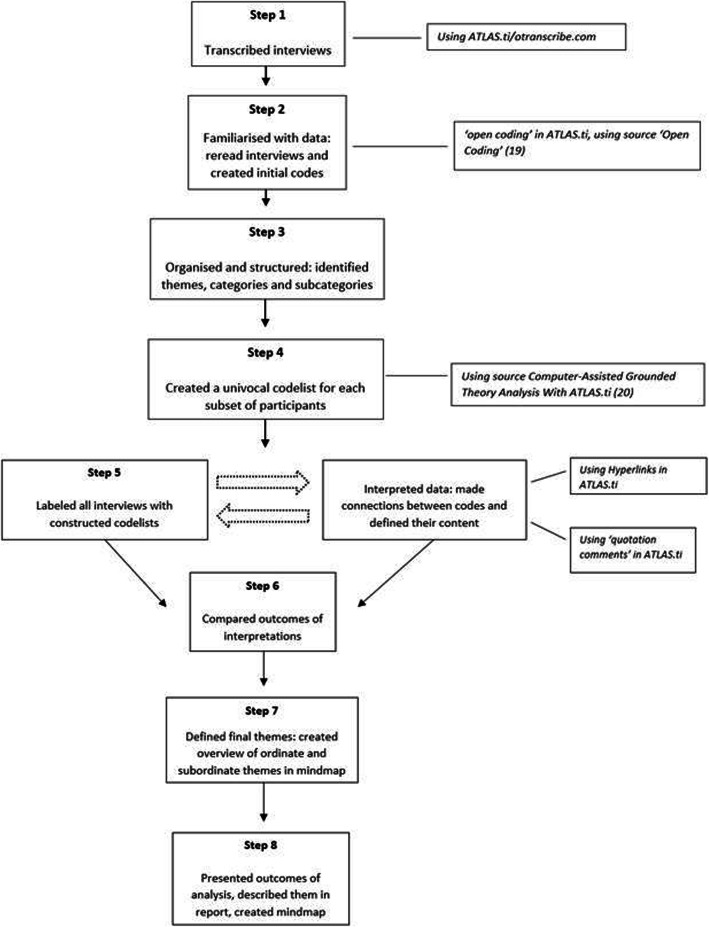
Phases of data analysis [19, 20]

Interviews were recorded and transcribed verbatim and a theory-driven thematic analysis was performed [19, 21]. The data-analysis was performed using ATLAS.ti (version 8.4 by Scientific Software Development GmbH) [22, 23]. The process involved eight phases as presented in Fig. 1. After getting familiarized with the data, the themes and categories were identified. The interviews were labelled in a structured manner with a built univocal code list. Simultaneously, the objective codes were refined by adding notes in the comment section and identifying connections between the codes and their categories. Throughout the process, themes, categories and codes were refined, added and merged. The interpreted outcomes of the interviews were compared within the subsets of participants and a map of the final themes was constructed. Lastly, a report of the thematic analysis was written.

## Results

**Fig. 2:**
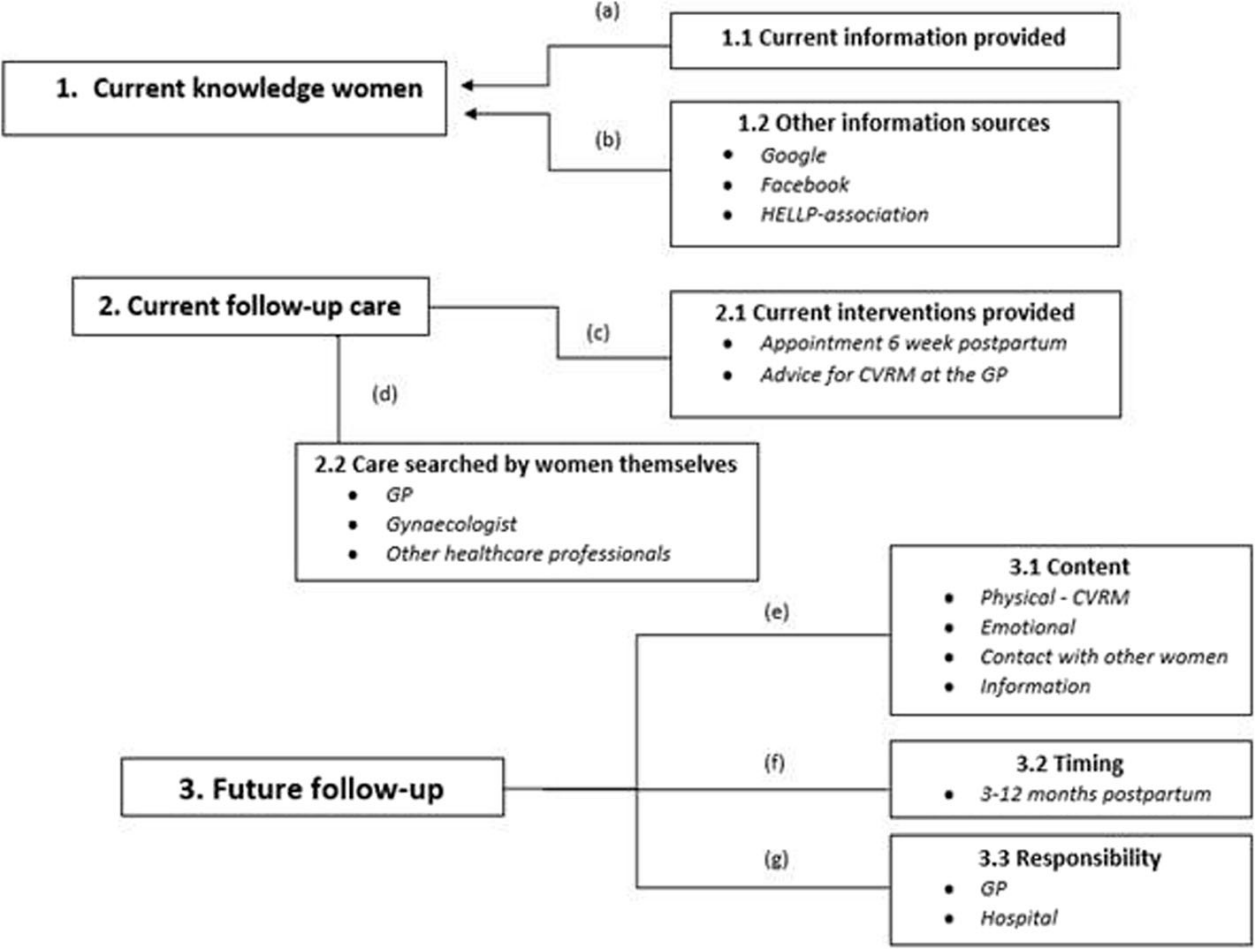
Overview of the main themes and the underlying associations that emerged from the interviews conducted with women with a preeclampsia history

**Fig. 3:**
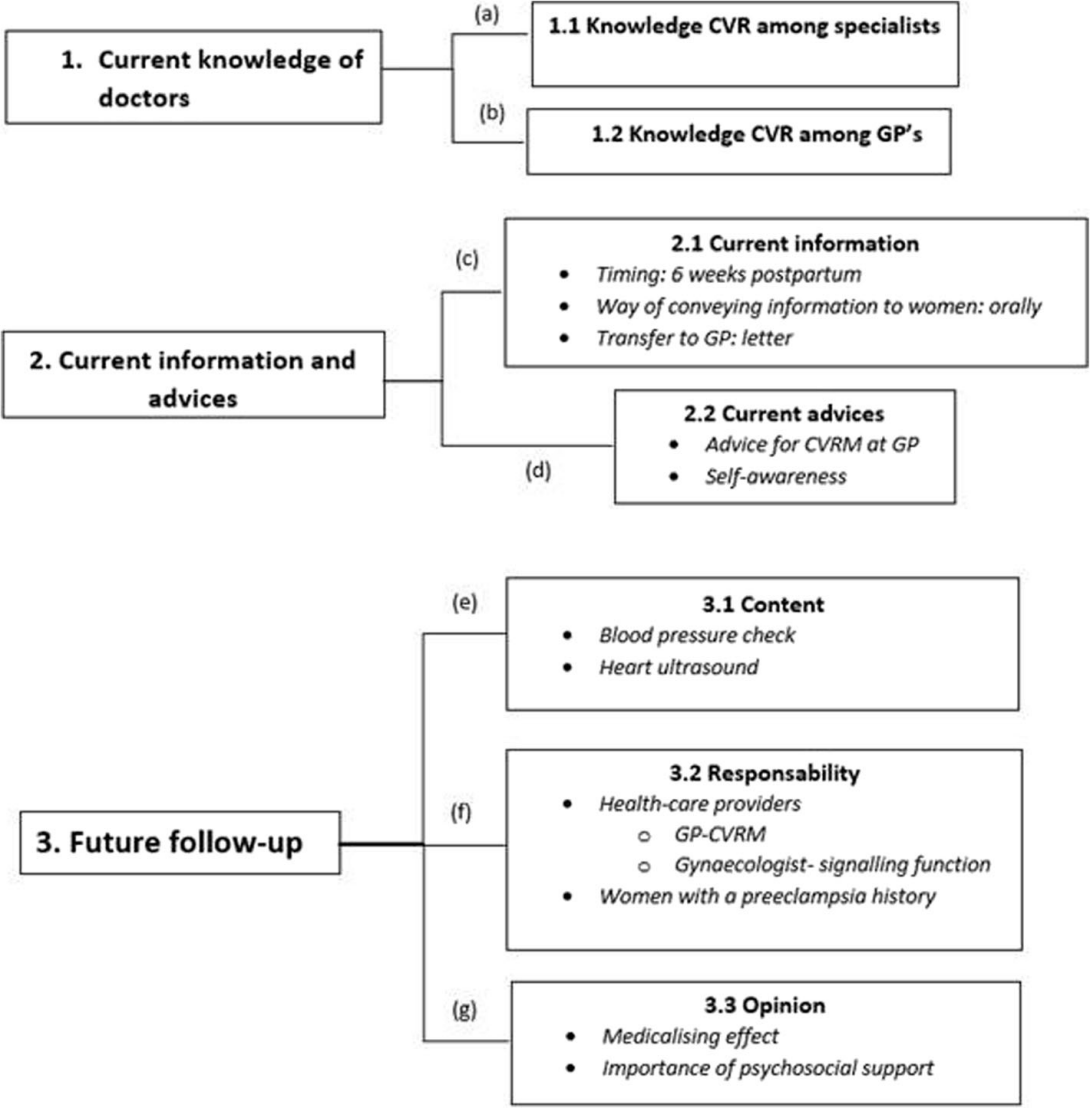
Overview of the main themes and the underlying associations that emerged from the interviews conducted with physicians

The sections below describe the perception of both women and physicians concerning the follow-up care for preeclampsia. Figures 2 and 3 outline the following themes: current knowledge and information, current follow-up, and future follow-up and their associations which are referred to as [a-g]. The patients interviewed were aged between 25 and 45 years. Seven women experienced early-onset PE (before the 34th week), seven women experienced late-onset PE (after the 34th week) [3], and one woman started showing symptoms right after delivery. Four out of the fifteen women experienced a pregnancy complicated with preeclampsia twice. An overview of participant characteristics can be found in Table 1.

**Table 1:** Participant characteristics *n* = 29

***Characteristic***	***n***
Patients experienced PE once	11
Patients experienced PE twice	4
General practitioners	4
Obstetricians	5
Cardiologists	2
Internal medicine specialists	3

### Patient section

#### Current knowledge and information

Women had some knowledge about their increased CVR following preeclampsia. Many, however, demonstrated a limited understanding of their personal risk and experienced a lack of information provided on the subject.

*“Maybe they did tell me afterwards, but a lot of things also went right past me.”*

Knowledge about preeclampsia was mostly gained using the internet, consulting Google and Facebook. Some women obtained their knowledge from the HELLP-foundation, whereas others consulted a healthcare professional for information, engaged in research or attended information days. Part of the women acquired information by means of a broadcast on television or in a newspaper*.* The information about the CVR was surprising and worrisome for some women.

*“Yes on websites, on Facebook groups, that kind of stuff.”*

*“I did start googling at some point.”*

*“That scared me a bit”*

The majority of women did intend to act on the potential risk. A healthy diet and exercise were mentioned as lifestyle adaptations some women implemented or continued after pregnancy. However, many women were either not putting lifestyle changes into practice or they did not have the tools to do so. A few women thought they had no influence on their personal CVR.

*“I thought like well, I should just do more with this, so I started to exercise more.”*

*“Then I was like, should I do something with this or not? But I still did not do anything with that.”*

#### Current follow-up

The only care provided after preeclampsia was an appointment 6 weeks postpartum, which felt insufficient for most women. Firstly, this appointment mainly focused on the delivery, resulting in a lack of time to discuss possible consequences of preeclampsia. Secondly, women pointed out that the appointment was too early after the pregnancy to be able to discuss future consequences and to comprehend all information given.

*“No just about the wound. He checked the wound and then we could go already because everything else was for the GP.”*

A small number of women were offered additional follow-up care, including contact with social work, a midwife, a psychologist, or a GP. Advice about future checks at the GP was given by some obstetricians, but the advice was inconsistent.

*“I had to check my blood pressure every year at the GP.”*

Some women sought care on their own initiative, predominantly at their GP. Others contacted an obstetrician or consulted other healthcare providers, such as a physiotherapist, haptotherapist, psychologist, dietician, shiatsu therapist, an osteopath and acupuncturist.

*“So on my request my cholesterol and glucose etc. are measured each year.”*

*“But I also went to a psychologist to do psychosomatic therapy.”*

#### Future follow-up

With regards to follow-up, the majority of women highlighted the importance of attention for the emotional impact of preeclampsia besides physical checks*.* Needing professional support for acceptance and processing of preeclampsia was often mentioned. In contrast, other women considered focus on the physical aspects more important than emotional support*.* Women mentioned receiving information about preeclampsia and the additional long-term consequences and risks as an essential component of the follow-up.

*“When life is getting back to normal you should get information like hey, you have to listen this can still have an aftermath.”*

Concerning the increased CVR, women pointed out they would prefer regular checks, such as blood pressure measurements and blood tests, especially if this would be offered by a healthcare professional. In general, women were motivated to make changes in their lifestyle if being advised so by healthcare professionals. For many women, lifestyle coaching would be of added value in adopting and maintaining a healthy lifestyle [2e].

*“I would prefer to get a phone call for a blood pressure check-up and that there is just a standard protocol for after your pregnancy.”*

*“Interviewer: If advice about adapting a healthy lifestyle is given, would you be willing to act on it?” “Yes,yes yes yes, absolutely. That is simply very important anyway.”*

There was no univocal answer on the preferred timing of the follow-up appointments concerning the consequences of the preeclampsia, but almost all women emphasized the importance of enough time between the delivery and the first appointment. Most women cited a time between 3 and 12 months postpartum as a good moment to address their own health and discuss the consequences including the increased CVR.

*“After 6 months you might start to recognize yourself and get some energy again and then you also get room for questions and for new information I think.”*

Regarding subsequent appointments, the preferred timing varied from multiple times a year to a frequency of every couple of years. Women indicated that the severity of the disorder, the sequelae and the personal needs regarding follow-up should be leading in the decision about the amount and timing of further appointments [2f].

*“Once every six months, in the beginning more regularly and then less frequent over the years, but that at least you have the attention on it’.*

The majority of women stated that contact with women who have had similar experiences would be of additional value, especially when being combined with information from professionals. In general, women would like to see the option being offered by the hospital.

*“What I would also really like is a uhm, for example talking to other women.”*

*“You could actively look for it yourself, but I think that it would be a good thing if a hospital would offer that.”*

There was no clear preference as to whom should take responsibility for the follow-up. Some women preferred the follow-up at the hospital because a medical specialist would have more specific knowledge. Other women indicated they would prefer to have CVRM at the GP for practical reasons. Women also suggested combining the two by having the first few appointments at the obstetrician followed by a transfer to the GP for long-term CVRM, provided that the communication goes well*.*

*“But maybe a follow-up conversation at an obstetrician and then the further follow-up just at the GP indeed.”*

### Physician section

#### Current knowledge CVR

The majority of the specialists mentioned there is some knowledge about the increased CVR among women with preeclampsia in their medical history within their specialties*.* Nevertheless, several GPs and cardiologists mentioned that this knowledge is fairly basic and insufficient when looking at the risk stratification in this large group of women. One GP stated to know nothing about the increased CVR. The lack of knowledge among GPs could partly be explained by the low prevalence in the general practice.

*“I think that everyone knows that there is an increased risk but the difficulty is, is that we don’t really know what we can do to decrease that risk”. (obstetrician)*

*“I actually don’t know so much about that” (GP)*

#### Current information and advice from physicians

Little information about the CVR was given by most obstetricians during the 6 weeks postpartum appointment. The information given was often inconsistent. Moreover, the information was often only orally provided. The specialists sometimes informed the GP by a letter, which advised monitoring blood pressure on a regular basis*.*

Part of the obstetricians advised women to go to their GP and have their blood pressure checked regularly. An internal medicine specialist emphasized that it is especially important that women themselves are aware of their increased risk*.*

*“You discuss all these things and you put it in the letter, which goes to the GP. (obstetrician)*

*“Well yes what would really help is if there would be a letter about a patient who has preeclampsia, and that the hospital sends a protocol with this letter with the warning ‘hey, pay attention to this patient’” (GP)*

#### Future follow-up

The majority of physicians would like to see follow-up in the form of a blood pressure check. In addition, a cardiologist mentioned he would also like to see an ultrasound of the heart. Additionally, physicians emphasized the importance of possible psychosocial consequences of experiencing preeclampsia.

*“They should always make sure to measure the blood pressure, either here or at a GP.” (obstetrician)*

*“But intuitively I would say that yes, there should be a follow-up for the high blood pressure.” (cardiologist)*

*“More important, I think, is the psychosocial risk that they have and I think this is demonstrated and that this group of people is much more, much earlier should be treated with EMDR [Eye movement desensitization and reprocessing], so in particular that psychosocial part. I think a lot can be gained there and uhm, and that you will certainly point that out, and that it could come back sometime”. (obstetrician)*

According to most physicians the follow-up can easily take place at the GP, possibly implemented as a part of the CVRM program. Some specialists thought there also needs to be a role for the hospital in the early stages of the follow-up. It is agreed upon that obstetricians should have a ‘signalling’ function, and that they should add a ‘preeclampsia flag’ to the woman’s medical file and transfer the patient to their GP for CVRM. In addition, many specialists believed a large part of the responsibility lies with the woman herself, provided that she is well informed. Physicians thought women are currently not sufficiently aware of their elevated CVR.

*“Well I think it would be the best if it could take place in primary care” (internal medicine specialist)*

*“Look, it would be the easiest at the GP. That is the most accessible and an annual check-up is fine according to me” (GP)*

*“The responsibility then lies with the women to do something with it” (cardiologist)*

A number of physicians warned for medicalization. Since it concerns a group of young women, it is important not to overburden them by means of an extensive follow-up. Lastly, obstetricians highlighted the importance of psychosocial support for these women.

*“But of course this is about healthy, young women. Who we would then already medicalize.”(GP)*

## Discussion

In this study, women showed basic knowledge regarding the increased CVR following preeclampsia and desired more information from health professionals regarding this topic, which is in line with previous literature [18, 24]. Knowledge about the increased CVR among physicians differed. Not all GPs had this knowledge. Although most specialists seem to have knowledge on the link between preeclampsia and CVR, the information and follow-up care they provided was often insufficient or inconsistent [17, 25–27].

Two main findings concerning the follow-up of women who suffered from preeclampsia emerged from our study: the need for improvement of the CVR follow-up and the desire for emotional support as part of the follow-up. Most women in our study pointed out a wish for blood pressure check-ups offered by the hospital or their GP, provided that their GP is well informed. Physicians supported this wish and stated that CVR follow-up of these women can easily be implemented as a part of the CVRM program at the GP, as it was already recommended by current Dutch guidelines [8].

Some physicians remained reluctant to provide information and interventions because recommendations concerning the long-term CVRM following preeclampsia are inconsistent. Additionally, physicians stressed the importance of avoiding medicalization.

As for timing, a Dutch study suggested that gynaecologists should discuss the CVR following preeclampsia and provide information at the regular visit 6 weeks postpartum [9]. Based on the opinion of the interviewed women and previous studies we propose that a later moment, between 3 and 12 months postpartum, would be more suitable to address these consequences and following interventions [18, 28]. The optimal timing and content of this information must be explored in further studies, as there are inconsistencies in current literature.

Since we observed differences in knowledge and awareness among GPs, we suggest that obstetricians should have a signalling function by writing a clear referral letter to the GP which states the preeclampsia history and recommendation to include the patient into their CVRM. Given the known association between lifestyle and CVD, lifestyle interventions should be part of this CVRM [9, 29]. Women are motivated to make changes in their lifestyle and pointed out that receiving counselling would be beneficial for adopting and maintaining a healthy lifestyle, which is supported by numerous studies [9, 18, 30, 31]. When women do not have the time or feel the need for a follow-up at the GP, there is the option to provide them with instructions for measuring the blood pressure at home. Advice regarding the instrument reliability and interpretations of results should be given, as well as adequate information on when to contact the caregiver.

In addition to a follow-up focusing on the physical consequences of preeclampsia, women reported a desire for emotional support as they currently experience a lack of recognition and support from their surroundings concerning the physical and psychosocial consequences. Although this study did not focus on the psychosocial consequences of PE, this emerged as an important theme in the majority of the interviews. Obstetricians supported the importance of emotional support for these women but it has not yet been implemented in current practice. It has been assumed that improving the social support can be achieved by creating more awareness for this topic among physicians and by providing opportunities for contact with other patients [30, 32]. In addition to individual counselling, group counselling might be suitable to meet the desire for recognition and create an opportunity for healthcare professionals to provide information.

Implementing these recommendations can be the first step in improving the current follow-up care for women with a pregnancy complicated by preeclampsia. More in-depth research with larger numbers of participants should be conducted to substantiate our findings, since this was a broad exploratory study which served to create an overview of the most important shortcomings of the current strategy. Future studies can clarify which subsets of women with a history of preeclampsia would need follow-up or even more thorough follow-up. Eventually an unambiguous evidence-based guideline regarding the follow-up for women with a pregnancy complicated by preeclampsia should be developed. This guideline should be evaluated in terms of patient acceptability, clinical feasibility and effectivity.

### Strengths and limitations

Selection bias may have occurred in this study since all women were recruited via de HELLP foundation, and thus may have already had above-average interest in their health. In addition, recall bias may have occurred as the amount of time that passed since having had PE differed amongst the interviewed women. Furthermore, we interviewed a group of physicians by including only specialists who are currently working at the UMCG. Although our interviews and analyses were carried out by at least two researchers, investigator bias may have arisen as this is inherent to qualitative research. Another limitation is the lack of background- and demographic information of the participants, including educational level and former health status. The broad setup of our study can be regarded as a strength. Since we interviewed physicians with different professions and women with a variety of age, years postpartum and severity of the disorder, we studied the topic from many perspectives.

Additionally, by following a semi-structured style of interviewing, the participants were free to express their own views and opinions whilst the interviewers were able to keep the subjects relevant. This lead to unexpected findings, such as the importance of a psychosocial aspect in the follow-up, that we as researchers had not yet identified as paramount.

## Conclusions

There is limited knowledge on the association between preeclampsia and the increased risk of future cardiovascular disease among women and physicians who participated in this study. Women voiced a general wish for a more extensive follow-up in the future. They ideally want a follow-up after a pregnancy complicated by preeclampsia to consist of information provided by physicians, emotional support and regular check-ups including lifestyle counselling. Physicians remain reluctant in providing extensive follow-up because of inconsistent recommendations and guidelines, as well as a lack of evidence-based research concerning this topic. Introducing these women into the standardized CVRM at the GP could be the most appropriate strategy at this moment to decrease their chance of future cardiovascular events.

## Supplementary information

**Additional file 1.** Interviews for women and physicians - translated from Dutch.

## Data Availability

Data will be available from the corresponding author upon resendable request.

## Abbreviations

CVD: Cardiovascular disease
CVR: Cardio vascular risk
CVRM: Cardiovascular risk management
GP: General practitioner
HDP: Hypertensive disorders of pregnancy
