# Supplementary material for: Investigating the current knowledge and needs concerning a follow-up for long-term cardiovascular risks in Dutch women with a preeclampsia history: a qualitative study
Source: BMC Pregnancy Childbirth. 2020 Aug 24;20:486. doi: 10.1186/s12884-020-03179-1 (PMC7444252; doi:10.1186/s12884-020-03179-1)
Supplement: Supplementary file 1 — Additional file 1. Interviews for women and physicians - translated from Dutch. [file 12884_2020_3179_MOESM1_ESM.docx]

Interviews for women and physicians - translated from Dutch

***Interviews for Physicians***

**1. Baseline characteristics – all physicians**

- How long have you been practicing this specialty?
- Where have you worked before?
  - Can you describe your past jobs?
- Where have you studied medicine?
- Has your employer/the hospital shaped your opinion on this topic? If yes, in what way?

**2. Interview for obstetricians**

***Knowledge and Information***

- What do you know about the cardiovascular risk in women who have had preeclampsia?
- If and to what extent do you think knowledge about the increased cardiovascular risk in these women exists within your specialism?
  - Do you think there should be more attention for this topic within your specialty?
- How would you describe the knowledge of women about their increased cardiovascular risk after experiencing preeclampsia?
- What kind of information do you provide for women who have had preeclampsia concerning their cardiovascular risk?
  - In what way do you provide this information?
  - When in the treatment trajectory do you provide the information?
  - How is this information received by the patient, in general?
  - In your opinion, which way of conveying information would be most ideal?

***Advices and interventions***

- What kind of advice do you give to a woman who has had preeclampsia concerning their cardiovascular risk?
- Which type of interventions or additional investigations do you perform in women who carry this risk?
  - Do you refer to another medical specialist?
- In what way do you involve the GP of the woman who has had preeclampsia?
  - Do you send a letter with recommendations or recommend follow-up?

***Type of follow-up***

- What would be the ideal follow-up for these women, in your opinion?
  - Whose job/responsibility should this be?
  - Would you involve the GP?
  - Do you think there should be a standardized guideline specifically for these women with an increased cardiovascular risk after preeclampsia?
  - What would still be necessary in order to facilitate your preferred follow-up in the north of the Netherlands?

***Opinion on the approach of the Erasmus Medical Center, Rotterdam (see below)***

- What is your opinion on the approach of the Erasmus MC? How would you visualize such a (comparable) follow-up in the north of the Netherlands?

**3. Interview general practitioners**

***Knowledge and Information***

- What do you know about the cardiovascular risk in women who have had preeclampsia?
- If and to what extent do you think knowledge about the increased cardiovascular risk in these women exists within your specialty?
  - Do you think there should be more attention for this topic within your specialty?
- How would you describe the knowledge of women about their increased cardiovascular risk after experiencing preeclampsia?
- What kind of information do you provide for women who have had preeclampsia concerning their cardiovascular risk?
  - In what way do you provide this information?
  - How is this information received by the patient, in general?

***Advices and Interventions***

- Do you ask female patients about their pregnancies when they come to your office with cardiovascular complaints?
  - How often do you ask this, approximately?
- How often do you check the blood pressure in women who have had preeclampsia?
  - What are your considerations for doing/not doing this?
- What kind of advice do you provide to women who have had preeclampsia concerning the cardiovascular risk?
- What kind of interventions or additional investigations do you perform in women with this risk?
- Is a follow-up and/or additional investigations necessary in these women, in your opinion, and why?
- What would be the ideal follow-up for these women, in your opinion?
  - Whose job/responsibility should this be?
  - How would you like to see the cooperation between GP and other specialists in this area?
  - Do you think there should be a standardized guideline specifically for these women with an increased cardiovascular risk after preeclampsia?
  - What would still be necessary in order to facilitate your preferred follow-up in the north of the Netherlands?

***Opinion on the approach of the Erasmus Medical Center, Rotterdam (see below)***

- What is your opinion on the approach of the Erasmus MC? How would you visualize such a (comparable) follow-up in the north of the Netherlands?

**4. Interview internal / vascular medicine specialists**

***Knowledge and information***

- What do you know about the cardiovascular risk in women who have had preeclampsia?
- If and to what extent do you think knowledge about the increased cardiovascular risk in these women exists within your specialty?
  - Do you think there should be more attention for this topic within your specialty?
- How would you describe the knowledge of women about their increased cardiovascular risk after experiencing preeclampsia?
- What kind of information do you provide for women who have had preeclampsia concerning their cardiovascular risk?
  - In what way do you provide this information?
  - How is this information received by the patient, in general?

***Advices and interventions***

- Do you ask female patients about their pregnancies when they come to your outpatient clinic?
  - How often do you ask this, approximately?
- What kind of advice do you provide to women who have had preeclampsia concerning the cardiovascular risk?
- What kind of interventions or additional investigations do you perform in women with this risk? What are your considerations?

***Type of follow-up***

- Is a follow-up and/or additional investigations necessary in these women, in your opinion, and why?
- What would be the ideal follow-up for these women, in your opinion?
  - In what way could your specialty contribute to healthcare for these women?
  - Whose job/responsibility should that be?
  - Would you involve the GP, if yes, how?
  - Do you think there should be a standardized guideline specifically for these women with an increased cardiovascular risk after preeclampsia?
  - What would still be necessary in order to facilitate your preferred follow-up in the north of the Netherlands?

***Opinion on the approach of the Erasmus Medical Center, Rotterdam (see below)***

- What is your opinion on the approach of the Erasmus MC? How would you visualize such a (comparable) follow-up in the north of the Netherlands?

***Interview for women***

***1. Baseline characteristics***

- What is your age?
- In which city/village do you live?
- What was your age at the time of the birth of your child (after the pregnancy complicated by preeclampsia)?
- How many times have you been pregnant?
- In which week of the pregnancy did you get preeclampsia?
- At which hospital were you treated for your preeclampsia?
- What was the birth weight of your child?
- Did you use medicaments during the pregnancy, and if yes, which ones?
- Do you currently use antihypertensive drugs?

***2. Knowledge***

*What do women know about possible (long-term) consequences / risks of pre-eclampsia and how did they obtain this information?*

- What do you know about the increased long-term risk of cardiovascular diseases, due to preeclampsia?
  - How did you get this information?
  - What do you think of the way you obtained this information?
- What information did you receive from your physician at the time about the consequences and aftercare of your preeclampsia?
  - What do you think about the information you have obtained?
- Do you think you received sufficient information?
- Do you feel that all your questions have been answered?
- How did and does this information about this increased risk of cardiovascular disease affect you?
  - Are you concerned about possible consequences?
  - Are you doing something with this information?
- What do you think you can do yourself to limit long-term consequences?

***3. Interventions***

To what extent were interventions offered to you/was there some sort of follow-up?

- What kind of check-ups or follow-up care did you receive after delivery?
  - How often were these check-ups? After how many weeks or months?
  - What was discussed/done during the check-up?
- Does your doctor or GP pay attention to PE in your medical history?
- What is being done or discussed?
  - What do you think of these interventions/recommendations?
  - Do you think these recommendations are useful?
  - Do you follow these recommendations?

***4. Future***

‘What would women prefer regarding the follow-up/after care?’

- What do you think about the amount of attention doctors currently pay to the consequences of preeclampsia?
- Do you feel the need for more follow-up treatments/more check-ups?
  - If yes > In what way do you prefer to see this?
    - How often would you want to have a check-up?
    - Would you prefer to have these check-ups at your GP or in the hospital/with a gynaecologist?
  - If no > why not?
- What do you think of Erasmus hospital’s approach? (give a small introduction about the Erasmus approach prior to this question)
- How do you feel about changing your lifestyle to limit possible consequences?
  - What motivates you?
  - What holds you back to make these changes in your lifestyle?
  - What do you think would help you to make these changes?

*Approach Erasmus Medical Center*

*The Erasmus Medical Center has an extensive follow-up for women who have had severe preeclampsia or HELLP. They will be reviewed at 6 weeks, 3 months and 1 year postpartum, after which they will be reviewed every 2 years. During this out-patient clinic check-up blood values, blood pressure and overall health status/condition will be discussed. These out-patient clinic appointments are done by an internal medicine specialist together with a gynaecologist. Women also take a small test about their lifestyle after which they will get some recommendations about this. Sometimes the thickness of the carotid arteries is measured. If necessary, interventions are suggested to prevent cardiovascular diseases.*

----------------------------------------------------------end of interviews----------------------------------------------------------
